# 1-(6-Fluoro-1,3-benzothia­zol-2-yl)-2-(1-phenyl­ethyl­idene)hydrazine

**DOI:** 10.1107/S1600536812030851

**Published:** 2012-07-14

**Authors:** Hoong-Kun Fun, Ching Kheng Quah, D. Munirajasekhar, M. Himaja, B. K. Sarojini

**Affiliations:** a X-ray Crystallography Unit, School of Physics, Universiti Sains Malaysia, 11800 USM, Penang, Malaysia; b Chemistry Division, School of Advanced Sciences, VIT University, Vellore 632 014, Tamil Nadu, India; c Department of Chemistry, P. A. College of Engineering, Nadupadavu, Mangalore 574 153, India

## Abstract

The asymmetric unit of the title compound, C_15_H_12_FN_3_S, consists of two independent mol­ecules with comparable geometries. In one mol­ecule, the 1,3-benzothia­zole ring system (r.m.s. deviation = 0.011 Å) forms a dihedral angle of 19.86 (6)° with the phenyl ring. The corresponding r.m.s. deviation and dihedral angle for the other mol­ecule are 0.014 Å and 22.32 (6)°, respectively. In the crystal, mol­ecules are linked *via* N—H⋯N, C—H⋯F and C—H⋯N hydrogen bonds into a three-dimensional network. The crystal studied was a non-merohedral twin with a refined BASF value of 0.301 (2).

## Related literature

For general background to and the biological activities of benzothia­zoles derivatives, see: Al-Soud *et al.* (2006 ▶); Kini *et al.* (2007 ▶); Munirajasekhar *et al.* (2011 ▶); Gurupadayya *et al.* (2008 ▶); Bowyer *et al.* (2007 ▶); Mittal *et al.* (2007 ▶); Pozas *et al.* (2005 ▶); Rana *et al.* (2008 ▶). For standard bond-length data, see: Allen *et al.* (1987 ▶). For the stability of the temperature controller used for the data collection, see: Cosier & Glazer (1986 ▶).
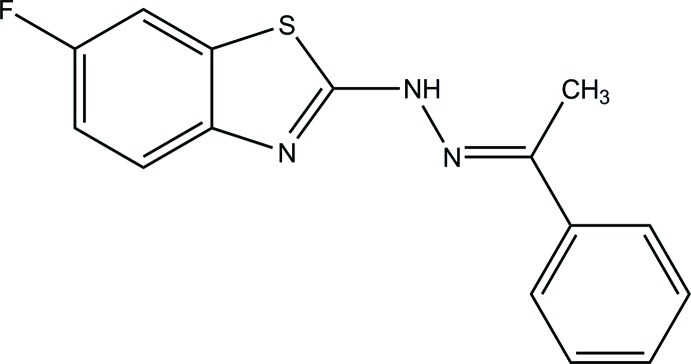


## Experimental

### 

#### Crystal data

C_15_H_12_FN_3_S
*M* *_r_* = 285.34
Monoclinic, 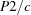

*a* = 28.312 (3) Å
*b* = 7.2952 (7) Å
*c* = 13.0626 (13) Å
β = 103.151 (2)°
*V* = 2627.2 (5) Å^3^
*Z* = 8
Mo *K*α radiation
μ = 0.25 mm^−1^
*T* = 100 K
0.46 × 0.21 × 0.14 mm

#### Data collection

Bruker SMART APEXII DUO CCD area-detector diffractometer
Absorption correction: multi-scan (*SADABS*; Bruker, 2009 ▶) *T*_min_ = 0.894, *T*_max_ = 0.965
52781 measured reflections
7411 independent reflections
7049 reflections with *I* > 2σ(*I*)
*R*_int_ = 0.036

#### Refinement

*R*[*F* ^2^ > 2σ(*F* ^2^)] = 0.033
*wR*(*F* ^2^) = 0.080
*S* = 1.06
7411 reflections
364 parameters
H-atom parameters constrained
Δρ_max_ = 0.46 e Å^−3^
Δρ_min_ = −0.43 e Å^−3^

### 

Data collection: *APEX2* (Bruker, 2009 ▶); cell refinement: *SAINT* (Bruker, 2009 ▶); data reduction: *SAINT*; program(s) used to solve structure: *SHELXTL* (Sheldrick, 2008 ▶); program(s) used to refine structure: *SHELXTL*; molecular graphics: *SHELXTL*; software used to prepare material for publication: *SHELXTL* and *PLATON* (Spek, 2009 ▶).

## Supplementary Material

Crystal structure: contains datablock(s) global, I. DOI: 10.1107/S1600536812030851/rz2786sup1.cif

Structure factors: contains datablock(s) I. DOI: 10.1107/S1600536812030851/rz2786Isup2.hkl

Supplementary material file. DOI: 10.1107/S1600536812030851/rz2786Isup3.cml

Additional supplementary materials:  crystallographic information; 3D view; checkCIF report

## Figures and Tables

**Table 1:** Hydrogen-bond geometry (Å, °)

*D*—H⋯*A*	*D*—H	H⋯*A*	*D*⋯*A*	*D*—H⋯*A*
N2*A*—H1N*A*⋯N1*A* ^i^	0.93	1.99	2.902 (2)	165
N2*B*—H1N*B*⋯N1*B* ^ii^	0.79	2.14	2.9184 (18)	168
C5*B*—H5*BA*⋯F1*B* ^iii^	0.95	2.51	3.310 (2)	142
C12*B*—H12*A*⋯F1*A* ^iv^	0.95	2.52	3.289 (2)	138
C12*A*—H12*B*⋯F1*B* ^v^	0.95	2.43	3.200 (2)	138
C15*B*—H15*A*⋯N1*B* ^ii^	0.98	2.57	3.503 (2)	160

Symmetry codes: (i) 
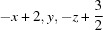
; (ii) 
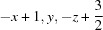
; (iii) 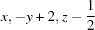; (iv) 
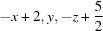
; (v) 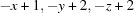.

**Fig. 1.**
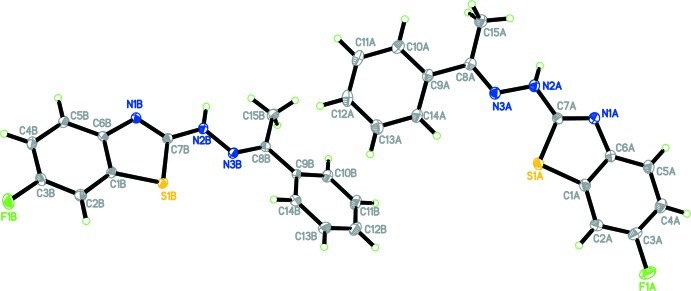
The molecular structure of the title compound showing 50% probability displacement ellipsoids for non-H atoms.

**Fig. 2.**
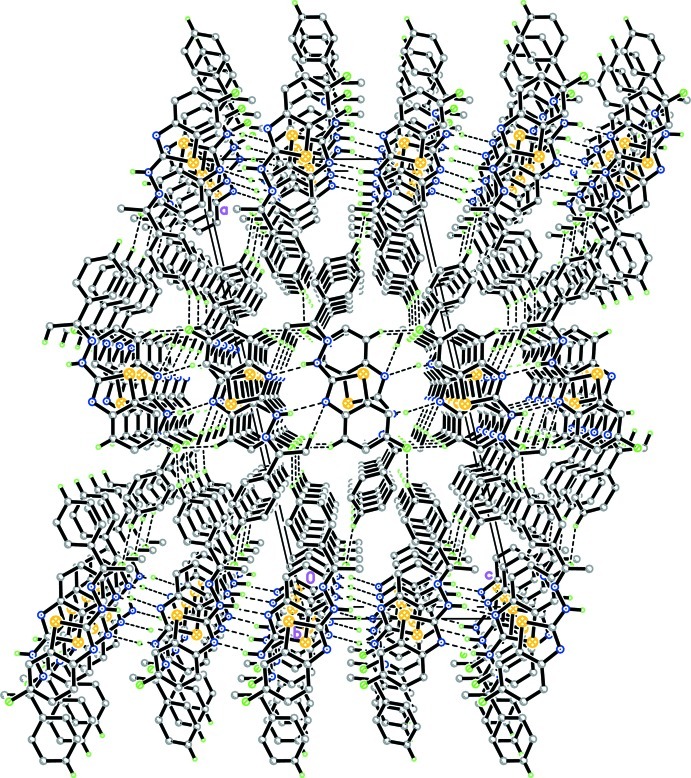
The crystal structure of the title compound, viewed along the b axis. H atoms not involved in hydrogen bonds (dashed lines) have been omitted for clarity.

## supplementary crystallographic information

### Comment

Benzothiazoles are very important bicyclic compounds which are of great interest because of their biological activities. The substituted benzothiazole derivatives have emerged as significant components in various diversified therapeutic applications. The literature review reveals that benzothiazoles and their derivatives show considerable activity including potent inhibition of human immunodeficiency virus type 1 (HIV-1) replication by HIV-1 protease inhibition (Al-Soud *et al.*, 2006), antitumor (Kini *et al.*, 2007), anthelmintic (Munirajasekhar *et al.*, 2011) analgesic and anti-inflammatory (Gurupadayya *et al.*, 2008), antimalarial (Bowyer *et al.*, 2007), antifungal (Mittal *et al.*, 2007), anticandidous activities (Pozas *et al.*, 2005) and various CNS activities (Rana *et al.*, 2008). The present work describes the synthesis and crystal structure of the title compound, 1-(6-fluoro1,3-benzothiazol-2-yl)-2-(1-phenylethylidene)hydrazine, which was prepared from the condensation reaction of 1-(6-fluoro1,3-benzothiazol-2-yl)hydrazine by refluxing for 2 h with acetophenone in presence of methanol.

The asymmetric unit (Fig. 1) of the title compound consists of two independent molecules (*A* and *B*), with comparable geometries. In molecule *A*, the 1,3-benzothiazol-2-yl ring system (S1A/N1A/C1A-C7A, r.m.s. deviation = 0.011 Å) forms a dihedral angle of 19.86 (6)° with the phenyl ring (C9A-C14A). The corresponding r.m.s. deviation and dihedral angle for molecule *B* are 0.014 Å and 22.32 (6)°, respectively. Bond lengths (Allen *et al.*, 1987) and angles are within normal ranges.

In the crystal structure, Fig. 2, molecules are linked *via* intermolecular N2A–H1NA···N1A, N2B–H1NB···N1B, C5B–H5BA···F1B, C12B–H12A···F1A, C12A–H12B···F1B and C15B–H15A···N1B hydrogen bonds (Table 1) into a three-dimensional network.

### Experimental

A mixture of 1-(6-fluoro1,3-benzothiazol-2-yl)hydrazine (1.83 g, 10 mmol) and acetophenone (1.2 g, 10 mmol) in methanol (50 mL) was refluxed at 2 h. After completion of the reaction, as monitored by TLC, the reaction mixture was poured into ice water (100 mL) whereby the crude product was precipitated as a yellow solid. The product obtained was washed with water and dried. The crude product was recrystalized from an ethylacetate/ethanol mixture (1:1 *v*/*v*). M.p.: 455-457 K.

### Refinement

The N-bound hydrogen atoms were located in a difference Fourier map and refined using a riding model with *U*_iso_(H) = 1.2 *U*_eq_(N) [N–H = 0.789 or 0.93 Å]. The remaining H atoms were positioned geometrically and refined using a riding model with C–H = 0.95 or 0.98 Å and *U*_iso_(H) = 1.2 or 1.5 *U*_eq_(C). A rotating-group model was applied for the methyl group. The crystal studied was a twin with twin law, 101 0-10 00-1 and BASF = 0.301 (2). Three outliers (-3 1 7; 2 1 0; 5 0 4) were omitted in the final refinement cycles.

### Crystal data

**Table:**

C_15_H_12_FN_3_S	*F*(000) = 1184
*M**_r_* = 285.34	*D*_x_ = 1.443 Mg m^−^^3^
Monoclinic, *P*2/*c*	Mo *K*α radiation, λ = 0.71073 Å
Hall symbol: -P 2yc	Cell parameters from 9959 reflections
*a* = 28.312 (3) Å	θ = 2.9–29.6°
*b* = 7.2952 (7) Å	µ = 0.25 mm^−^^1^
*c* = 13.0626 (13) Å	*T* = 100 K
β = 103.151 (2)°	Block, yellow
*V* = 2627.2 (5) Å^3^	0.46 × 0.21 × 0.14 mm
*Z* = 8	

### Data collection

**Table:**

Bruker SMART APEXII DUO CCD area-detector diffractometer	7411 independent reflections
Radiation source: fine-focus sealed tube	7049 reflections with *I* > 2σ(*I*)
Graphite monochromator	*R*_int_ = 0.036
φ and ω scans	θ_max_ = 29.7°, θ_min_ = 0.7°
Absorption correction: multi-scan (*SADABS*; Bruker, 2009)	*h* = −39→39
*T*_min_ = 0.894, *T*_max_ = 0.965	*k* = −10→10
52781 measured reflections	*l* = −18→18

### Refinement

**Table:**

Refinement on *F*^2^	Primary atom site location: structure-invariant direct methods
Least-squares matrix: full	Secondary atom site location: difference Fourier map
*R*[*F*^2^ > 2σ(*F*^2^)] = 0.033	Hydrogen site location: inferred from neighbouring sites
*wR*(*F*^2^) = 0.080	H-atom parameters constrained
*S* = 1.06	*w* = 1/[σ^2^(*F*_o_^2^) + (0.0333*P*)^2^ + 1.4375*P*] where *P* = (*F*_o_^2^ + 2*F*_c_^2^)/3
7411 reflections	(Δ/σ)_max_ = 0.002
364 parameters	Δρ_max_ = 0.46 e Å^−^^3^
0 restraints	Δρ_min_ = −0.43 e Å^−^^3^

### Special details

**Table:**

Experimental. The crystal was placed in the cold stream of an Oxford Cryosystems Cobra open-flow nitrogen cryostat (Cosier & Glazer, 1986) operating at 100.0 (1) K.
Geometry. All esds (except the esd in the dihedral angle between two l.s. planes) are estimated using the full covariance matrix. The cell esds are taken into account individually in the estimation of esds in distances, angles and torsion angles; correlations between esds in cell parameters are only used when they are defined by crystal symmetry. An approximate (isotropic) treatment of cell esds is used for estimating esds involving l.s. planes.
Refinement. Refinement of F^2^ against ALL reflections. The weighted R-factor wR and goodness of fit S are based on F^2^, conventional R-factors R are based on F, with F set to zero for negative F^2^. The threshold expression of F^2^ > 2sigma(F^2^) is used only for calculating R-factors(gt) etc. and is not relevant to the choice of reflections for refinement. R-factors based on F^2^ are statistically about twice as large as those based on F, and R- factors based on ALL data will be even larger.

### Fractional atomic coordinates and isotropic or equivalent isotropic displacement parameters (Å^2^)

**Table:**

	*x*	*y*	*z*	*U*_iso_*/*U*_eq_	
S1A	0.972160 (13)	0.76932 (5)	1.01810 (3)	0.01518 (8)	
F1A	1.12593 (4)	0.52213 (16)	1.27187 (9)	0.0279 (2)	
N1A	1.02856 (5)	0.76694 (18)	0.88368 (11)	0.0153 (2)	
N2A	0.94918 (4)	0.86931 (19)	0.81485 (11)	0.0164 (3)	
H1NA	0.9510	0.8461	0.7457	0.020*	
N3A	0.90434 (4)	0.88095 (18)	0.83979 (11)	0.0156 (2)	
C1A	1.03203 (5)	0.6981 (2)	1.06242 (13)	0.0157 (3)	
C2A	1.05463 (6)	0.6359 (2)	1.16183 (13)	0.0185 (3)	
H2AA	1.0381	0.6291	1.2174	0.022*	
C3A	1.10265 (6)	0.5845 (2)	1.17516 (13)	0.0197 (3)	
C4A	1.12836 (6)	0.5918 (2)	1.09697 (14)	0.0197 (3)	
H4AA	1.1615	0.5562	1.1112	0.024*	
C5A	1.10515 (5)	0.6519 (2)	0.99766 (13)	0.0176 (3)	
H5AA	1.1220	0.6561	0.9425	0.021*	
C6A	1.05651 (5)	0.7064 (2)	0.97977 (13)	0.0142 (3)	
C7A	0.98479 (5)	0.8033 (2)	0.89425 (12)	0.0141 (3)	
C8A	0.86838 (5)	0.9481 (2)	0.77176 (12)	0.0151 (3)	
C9A	0.82091 (5)	0.9398 (2)	0.80292 (13)	0.0154 (3)	
C10A	0.77909 (5)	1.0154 (2)	0.73955 (13)	0.0190 (3)	
H10B	0.7811	1.0814	0.6780	0.023*	
C11A	0.73430 (5)	0.9950 (2)	0.76586 (14)	0.0224 (3)	
H11B	0.7061	1.0482	0.7224	0.027*	
C12A	0.73052 (6)	0.8977 (2)	0.85482 (15)	0.0222 (3)	
H12B	0.6999	0.8819	0.8716	0.027*	
C13A	0.77206 (6)	0.8236 (2)	0.91916 (15)	0.0219 (3)	
H13B	0.7699	0.7578	0.9807	0.026*	
C14A	0.81679 (5)	0.8455 (2)	0.89382 (13)	0.0180 (3)	
H14B	0.8450	0.7957	0.9388	0.022*	
C15A	0.87077 (6)	1.0251 (2)	0.66642 (14)	0.0212 (3)	
H15D	0.9046	1.0273	0.6600	0.032*	
H15E	0.8577	1.1500	0.6599	0.032*	
H15F	0.8516	0.9481	0.6107	0.032*	
S1B	0.530131 (12)	0.72187 (5)	1.04747 (3)	0.01352 (8)	
F1B	0.37853 (3)	0.96714 (15)	1.16254 (8)	0.0242 (2)	
N1B	0.47380 (4)	0.75091 (17)	0.85933 (10)	0.0135 (2)	
N2B	0.55248 (4)	0.64677 (18)	0.86209 (10)	0.0154 (2)	
H1NB	0.5496	0.6729	0.8024	0.018*	
N3B	0.59655 (4)	0.60724 (17)	0.92759 (10)	0.0139 (2)	
C1B	0.47084 (5)	0.7988 (2)	1.03672 (12)	0.0125 (3)	
C2B	0.44856 (5)	0.8532 (2)	1.11663 (13)	0.0164 (3)	
H2BA	0.4652	0.8500	1.1885	0.020*	
C3B	0.40115 (5)	0.9118 (2)	1.08580 (13)	0.0161 (3)	
C4B	0.37534 (5)	0.9174 (2)	0.98252 (13)	0.0160 (3)	
H4BA	0.3425	0.9572	0.9656	0.019*	
C5B	0.39812 (5)	0.8640 (2)	0.90372 (13)	0.0154 (3)	
H5BA	0.3811	0.8676	0.8321	0.019*	
C6B	0.44636 (5)	0.80470 (19)	0.93059 (12)	0.0125 (3)	
C7B	0.51744 (5)	0.7067 (2)	0.91009 (12)	0.0131 (3)	
C8B	0.62998 (5)	0.5364 (2)	0.88735 (12)	0.0143 (3)	
C9B	0.67655 (5)	0.4959 (2)	0.96244 (12)	0.0133 (3)	
C10B	0.71024 (5)	0.3762 (2)	0.93491 (13)	0.0181 (3)	
H10A	0.7037	0.3245	0.8664	0.022*	
C11B	0.75312 (5)	0.3316 (2)	1.00643 (15)	0.0222 (3)	
H11A	0.7754	0.2487	0.9870	0.027*	
C12B	0.76319 (5)	0.4081 (2)	1.10577 (15)	0.0223 (3)	
H12A	0.7924	0.3780	1.1548	0.027*	
C13B	0.73027 (6)	0.5300 (2)	1.13402 (13)	0.0208 (3)	
H13A	0.7373	0.5832	1.2022	0.025*	
C14B	0.68728 (5)	0.5737 (2)	1.06267 (13)	0.0173 (3)	
H14A	0.6651	0.6570	1.0823	0.021*	
C15B	0.62514 (6)	0.4894 (2)	0.77311 (13)	0.0192 (3)	
H15A	0.5936	0.5318	0.7323	0.029*	
H15B	0.6276	0.3563	0.7655	0.029*	
H15C	0.6511	0.5496	0.7472	0.029*	

### Atomic displacement parameters (Å^2^)

**Table:**

	*U*^11^	*U*^22^	*U*^33^	*U*^12^	*U*^13^	*U*^23^
S1A	0.01302 (15)	0.01712 (18)	0.01631 (19)	0.00009 (13)	0.00523 (13)	−0.00027 (13)
F1A	0.0272 (5)	0.0317 (6)	0.0211 (5)	0.0061 (4)	−0.0024 (4)	0.0071 (5)
N1A	0.0138 (5)	0.0159 (6)	0.0158 (6)	0.0004 (5)	0.0027 (5)	0.0006 (5)
N2A	0.0130 (5)	0.0205 (6)	0.0160 (6)	0.0019 (5)	0.0040 (5)	0.0003 (5)
N3A	0.0135 (5)	0.0169 (6)	0.0166 (6)	0.0009 (4)	0.0039 (5)	−0.0002 (5)
C1A	0.0142 (6)	0.0144 (7)	0.0184 (7)	−0.0004 (5)	0.0038 (5)	0.0001 (6)
C2A	0.0204 (7)	0.0180 (7)	0.0173 (7)	−0.0005 (6)	0.0050 (6)	0.0018 (6)
C3A	0.0204 (7)	0.0177 (7)	0.0183 (8)	0.0017 (6)	−0.0012 (6)	0.0020 (6)
C4A	0.0156 (6)	0.0172 (7)	0.0248 (8)	0.0021 (5)	0.0020 (6)	−0.0002 (6)
C5A	0.0143 (6)	0.0160 (7)	0.0228 (8)	0.0007 (5)	0.0051 (6)	0.0003 (6)
C6A	0.0138 (6)	0.0122 (6)	0.0168 (7)	0.0000 (5)	0.0041 (5)	−0.0005 (5)
C7A	0.0148 (6)	0.0131 (6)	0.0148 (7)	−0.0014 (5)	0.0045 (5)	−0.0008 (5)
C8A	0.0150 (6)	0.0136 (7)	0.0167 (7)	0.0010 (5)	0.0033 (5)	−0.0021 (6)
C9A	0.0134 (6)	0.0130 (6)	0.0192 (7)	0.0010 (5)	0.0025 (5)	−0.0023 (6)
C10A	0.0177 (6)	0.0190 (7)	0.0192 (7)	0.0031 (6)	0.0017 (6)	−0.0005 (6)
C11A	0.0137 (6)	0.0231 (8)	0.0284 (9)	0.0046 (6)	0.0008 (6)	−0.0029 (7)
C12A	0.0151 (6)	0.0216 (8)	0.0310 (9)	0.0010 (6)	0.0073 (6)	−0.0037 (7)
C13A	0.0191 (7)	0.0200 (7)	0.0282 (9)	0.0011 (6)	0.0085 (6)	0.0008 (7)
C14A	0.0145 (6)	0.0176 (7)	0.0217 (8)	0.0022 (5)	0.0035 (6)	−0.0001 (6)
C15A	0.0192 (7)	0.0245 (8)	0.0204 (8)	0.0045 (6)	0.0056 (6)	0.0025 (6)
S1B	0.01062 (15)	0.01609 (17)	0.01314 (17)	0.00098 (12)	0.00124 (13)	0.00005 (13)
F1B	0.0191 (4)	0.0361 (6)	0.0192 (5)	0.0064 (4)	0.0078 (4)	−0.0052 (4)
N1B	0.0113 (5)	0.0168 (6)	0.0123 (6)	0.0002 (4)	0.0025 (4)	0.0001 (5)
N2B	0.0122 (5)	0.0198 (6)	0.0143 (6)	0.0038 (5)	0.0034 (5)	0.0009 (5)
N3B	0.0119 (5)	0.0146 (6)	0.0146 (6)	0.0007 (4)	0.0018 (4)	0.0011 (5)
C1B	0.0108 (5)	0.0133 (6)	0.0129 (6)	−0.0003 (5)	0.0016 (5)	−0.0014 (5)
C2B	0.0156 (6)	0.0201 (7)	0.0131 (7)	0.0000 (5)	0.0026 (5)	−0.0015 (6)
C3B	0.0158 (6)	0.0181 (7)	0.0161 (7)	0.0009 (5)	0.0069 (5)	−0.0027 (6)
C4B	0.0115 (6)	0.0163 (7)	0.0206 (7)	0.0016 (5)	0.0043 (5)	0.0008 (6)
C5B	0.0129 (6)	0.0165 (7)	0.0164 (7)	0.0008 (5)	0.0024 (5)	0.0012 (6)
C6B	0.0126 (6)	0.0125 (6)	0.0127 (6)	−0.0007 (5)	0.0036 (5)	0.0011 (5)
C7B	0.0130 (6)	0.0130 (6)	0.0127 (7)	−0.0006 (5)	0.0016 (5)	0.0000 (5)
C8B	0.0128 (6)	0.0146 (7)	0.0157 (7)	0.0005 (5)	0.0036 (5)	0.0016 (6)
C9B	0.0120 (6)	0.0139 (6)	0.0139 (7)	0.0003 (5)	0.0030 (5)	0.0028 (5)
C10B	0.0154 (6)	0.0175 (7)	0.0211 (8)	0.0019 (5)	0.0036 (6)	−0.0006 (6)
C11B	0.0153 (6)	0.0191 (7)	0.0313 (9)	0.0035 (5)	0.0032 (6)	0.0025 (7)
C12B	0.0154 (6)	0.0212 (8)	0.0275 (9)	−0.0008 (6)	−0.0009 (6)	0.0056 (7)
C13B	0.0189 (7)	0.0252 (8)	0.0167 (7)	−0.0023 (6)	0.0004 (6)	0.0021 (6)
C14B	0.0159 (6)	0.0200 (7)	0.0166 (7)	−0.0002 (5)	0.0047 (5)	0.0011 (6)
C15B	0.0190 (6)	0.0244 (8)	0.0140 (7)	0.0069 (6)	0.0032 (6)	−0.0016 (6)

### Geometric parameters (Å, º)

**Table:**

S1A—C1A	1.7414 (15)	S1B—C1B	1.7451 (15)
S1A—C7A	1.7521 (16)	S1B—C7B	1.7518 (16)
F1A—C3A	1.3632 (19)	F1B—C3B	1.3674 (17)
N1A—C7A	1.3051 (19)	N1B—C7B	1.3029 (18)
N1A—C6A	1.395 (2)	N1B—C6B	1.3976 (19)
N2A—C7A	1.3594 (19)	N2B—C7B	1.3608 (18)
N2A—N3A	1.3831 (17)	N2B—N3B	1.3735 (17)
N2A—H1NA	0.9319	N2B—H1NB	0.7882
N3A—C8A	1.2862 (19)	N3B—C8B	1.2906 (19)
C1A—C2A	1.387 (2)	C1B—C2B	1.394 (2)
C1A—C6A	1.411 (2)	C1B—C6B	1.403 (2)
C2A—C3A	1.383 (2)	C2B—C3B	1.379 (2)
C2A—H2AA	0.9500	C2B—H2BA	0.9500
C3A—C4A	1.384 (2)	C3B—C4B	1.381 (2)
C4A—C5A	1.385 (2)	C4B—C5B	1.389 (2)
C4A—H4AA	0.9500	C4B—H4BA	0.9500
C5A—C6A	1.401 (2)	C5B—C6B	1.3989 (19)
C5A—H5AA	0.9500	C5B—H5BA	0.9500
C8A—C9A	1.492 (2)	C8B—C9B	1.4831 (19)
C8A—C15A	1.502 (2)	C8B—C15B	1.507 (2)
C9A—C10A	1.395 (2)	C9B—C14B	1.396 (2)
C9A—C14A	1.400 (2)	C9B—C10B	1.399 (2)
C10A—C11A	1.395 (2)	C10B—C11B	1.391 (2)
C10A—H10B	0.9500	C10B—H10A	0.9500
C11A—C12A	1.386 (3)	C11B—C12B	1.381 (3)
C11A—H11B	0.9500	C11B—H11A	0.9500
C12A—C13A	1.390 (2)	C12B—C13B	1.397 (2)
C12A—H12B	0.9500	C12B—H12A	0.9500
C13A—C14A	1.389 (2)	C13B—C14B	1.391 (2)
C13A—H13B	0.9500	C13B—H13A	0.9500
C14A—H14B	0.9500	C14B—H14A	0.9500
C15A—H15D	0.9800	C15B—H15A	0.9800
C15A—H15E	0.9800	C15B—H15B	0.9800
C15A—H15F	0.9800	C15B—H15C	0.9800
			
C1A—S1A—C7A	87.80 (7)	C1B—S1B—C7B	88.19 (7)
C7A—N1A—C6A	109.10 (13)	C7B—N1B—C6B	109.69 (13)
C7A—N2A—N3A	113.78 (13)	C7B—N2B—N3B	115.76 (12)
C7A—N2A—H1NA	118.6	C7B—N2B—H1NB	117.1
N3A—N2A—H1NA	119.8	N3B—N2B—H1NB	122.8
C8A—N3A—N2A	119.03 (13)	C8B—N3B—N2B	118.47 (13)
C2A—C1A—C6A	121.90 (14)	C2B—C1B—C6B	121.68 (13)
C2A—C1A—S1A	128.01 (12)	C2B—C1B—S1B	128.46 (12)
C6A—C1A—S1A	110.08 (12)	C6B—C1B—S1B	109.84 (11)
C3A—C2A—C1A	115.95 (15)	C3B—C2B—C1B	116.47 (14)
C3A—C2A—H2AA	122.0	C3B—C2B—H2BA	121.8
C1A—C2A—H2AA	122.0	C1B—C2B—H2BA	121.8
F1A—C3A—C2A	117.48 (15)	F1B—C3B—C2B	117.68 (14)
F1A—C3A—C4A	118.20 (14)	F1B—C3B—C4B	118.38 (13)
C2A—C3A—C4A	124.32 (15)	C2B—C3B—C4B	123.93 (14)
C3A—C4A—C5A	119.11 (14)	C3B—C4B—C5B	118.94 (13)
C3A—C4A—H4AA	120.4	C3B—C4B—H4BA	120.5
C5A—C4A—H4AA	120.4	C5B—C4B—H4BA	120.5
C4A—C5A—C6A	119.00 (15)	C4B—C5B—C6B	119.48 (14)
C4A—C5A—H5AA	120.5	C4B—C5B—H5BA	120.3
C6A—C5A—H5AA	120.5	C6B—C5B—H5BA	120.3
N1A—C6A—C5A	125.09 (14)	N1B—C6B—C5B	125.34 (14)
N1A—C6A—C1A	115.19 (13)	N1B—C6B—C1B	115.16 (12)
C5A—C6A—C1A	119.71 (15)	C5B—C6B—C1B	119.49 (14)
N1A—C7A—N2A	123.27 (14)	N1B—C7B—N2B	123.47 (14)
N1A—C7A—S1A	117.84 (12)	N1B—C7B—S1B	117.10 (11)
N2A—C7A—S1A	118.87 (11)	N2B—C7B—S1B	119.42 (11)
N3A—C8A—C9A	114.63 (14)	N3B—C8B—C9B	115.75 (13)
N3A—C8A—C15A	125.59 (14)	N3B—C8B—C15B	125.78 (14)
C9A—C8A—C15A	119.75 (13)	C9B—C8B—C15B	118.47 (13)
C10A—C9A—C14A	118.33 (14)	C14B—C9B—C10B	118.65 (13)
C10A—C9A—C8A	121.20 (15)	C14B—C9B—C8B	120.69 (13)
C14A—C9A—C8A	120.36 (13)	C10B—C9B—C8B	120.65 (14)
C9A—C10A—C11A	120.53 (15)	C11B—C10B—C9B	120.98 (15)
C9A—C10A—H10B	119.7	C11B—C10B—H10A	119.5
C11A—C10A—H10B	119.7	C9B—C10B—H10A	119.5
C12A—C11A—C10A	120.59 (15)	C12B—C11B—C10B	119.90 (15)
C12A—C11A—H11B	119.7	C12B—C11B—H11A	120.0
C10A—C11A—H11B	119.7	C10B—C11B—H11A	120.0
C11A—C12A—C13A	119.33 (15)	C11B—C12B—C13B	119.84 (15)
C11A—C12A—H12B	120.3	C11B—C12B—H12A	120.1
C13A—C12A—H12B	120.3	C13B—C12B—H12A	120.1
C14A—C13A—C12A	120.21 (16)	C14B—C13B—C12B	120.24 (16)
C14A—C13A—H13B	119.9	C14B—C13B—H13A	119.9
C12A—C13A—H13B	119.9	C12B—C13B—H13A	119.9
C13A—C14A—C9A	120.99 (15)	C13B—C14B—C9B	120.37 (14)
C13A—C14A—H14B	119.5	C13B—C14B—H14A	119.8
C9A—C14A—H14B	119.5	C9B—C14B—H14A	119.8
C8A—C15A—H15D	109.5	C8B—C15B—H15A	109.5
C8A—C15A—H15E	109.5	C8B—C15B—H15B	109.5
H15D—C15A—H15E	109.5	H15A—C15B—H15B	109.5
C8A—C15A—H15F	109.5	C8B—C15B—H15C	109.5
H15D—C15A—H15F	109.5	H15A—C15B—H15C	109.5
H15E—C15A—H15F	109.5	H15B—C15B—H15C	109.5
			
C7A—N2A—N3A—C8A	177.41 (14)	C7B—N2B—N3B—C8B	174.69 (14)
C7A—S1A—C1A—C2A	−178.25 (16)	C7B—S1B—C1B—C2B	−177.91 (15)
C7A—S1A—C1A—C6A	0.09 (12)	C7B—S1B—C1B—C6B	0.62 (11)
C6A—C1A—C2A—C3A	0.6 (2)	C6B—C1B—C2B—C3B	0.4 (2)
S1A—C1A—C2A—C3A	178.81 (13)	S1B—C1B—C2B—C3B	178.78 (12)
C1A—C2A—C3A—F1A	−179.81 (14)	C1B—C2B—C3B—F1B	−179.57 (13)
C1A—C2A—C3A—C4A	0.0 (2)	C1B—C2B—C3B—C4B	0.6 (2)
F1A—C3A—C4A—C5A	178.95 (14)	F1B—C3B—C4B—C5B	179.14 (14)
C2A—C3A—C4A—C5A	−0.9 (3)	C2B—C3B—C4B—C5B	−1.0 (2)
C3A—C4A—C5A—C6A	1.0 (2)	C3B—C4B—C5B—C6B	0.4 (2)
C7A—N1A—C6A—C5A	178.88 (15)	C7B—N1B—C6B—C5B	178.95 (14)
C7A—N1A—C6A—C1A	0.23 (19)	C7B—N1B—C6B—C1B	−0.19 (18)
C4A—C5A—C6A—N1A	−178.99 (14)	C4B—C5B—C6B—N1B	−178.61 (14)
C4A—C5A—C6A—C1A	−0.4 (2)	C4B—C5B—C6B—C1B	0.5 (2)
C2A—C1A—C6A—N1A	178.25 (14)	C2B—C1B—C6B—N1B	178.26 (13)
S1A—C1A—C6A—N1A	−0.20 (17)	S1B—C1B—C6B—N1B	−0.39 (16)
C2A—C1A—C6A—C5A	−0.5 (2)	C2B—C1B—C6B—C5B	−0.9 (2)
S1A—C1A—C6A—C5A	−178.93 (12)	S1B—C1B—C6B—C5B	−179.59 (11)
C6A—N1A—C7A—N2A	178.47 (14)	C6B—N1B—C7B—N2B	179.28 (14)
C6A—N1A—C7A—S1A	−0.16 (17)	C6B—N1B—C7B—S1B	0.72 (16)
N3A—N2A—C7A—N1A	173.87 (14)	N3B—N2B—C7B—N1B	179.62 (14)
N3A—N2A—C7A—S1A	−7.51 (18)	N3B—N2B—C7B—S1B	−1.86 (18)
C1A—S1A—C7A—N1A	0.05 (13)	C1B—S1B—C7B—N1B	−0.81 (13)
C1A—S1A—C7A—N2A	−178.65 (13)	C1B—S1B—C7B—N2B	−179.43 (13)
N2A—N3A—C8A—C9A	175.07 (13)	N2B—N3B—C8B—C9B	−179.60 (13)
N2A—N3A—C8A—C15A	−2.9 (2)	N2B—N3B—C8B—C15B	−0.8 (2)
N3A—C8A—C9A—C10A	176.81 (14)	N3B—C8B—C9B—C14B	−16.3 (2)
C15A—C8A—C9A—C10A	−5.1 (2)	C15B—C8B—C9B—C14B	164.85 (14)
N3A—C8A—C9A—C14A	−7.2 (2)	N3B—C8B—C9B—C10B	162.52 (14)
C15A—C8A—C9A—C14A	170.95 (14)	C15B—C8B—C9B—C10B	−16.3 (2)
C14A—C9A—C10A—C11A	−0.8 (2)	C14B—C9B—C10B—C11B	1.5 (2)
C8A—C9A—C10A—C11A	175.31 (15)	C8B—C9B—C10B—C11B	−177.34 (15)
C9A—C10A—C11A—C12A	−0.6 (3)	C9B—C10B—C11B—C12B	−0.9 (2)
C10A—C11A—C12A—C13A	1.4 (3)	C10B—C11B—C12B—C13B	0.0 (3)
C11A—C12A—C13A—C14A	−0.6 (3)	C11B—C12B—C13B—C14B	0.3 (2)
C12A—C13A—C14A—C9A	−0.8 (3)	C12B—C13B—C14B—C9B	0.2 (2)
C10A—C9A—C14A—C13A	1.5 (2)	C10B—C9B—C14B—C13B	−1.1 (2)
C8A—C9A—C14A—C13A	−174.61 (15)	C8B—C9B—C14B—C13B	177.69 (14)

### Hydrogen-bond geometry (Å, º)

**Table:**

*D*—H···*A*	*D*—H	H···*A*	*D*···*A*	*D*—H···*A*
N2*A*—H1*NA*···N1*A*^i^	0.93	1.99	2.902 (2)	165
N2*B*—H1*NB*···N1*B*^ii^	0.79	2.14	2.9184 (18)	168
C5*B*—H5*BA*···F1*B*^iii^	0.95	2.51	3.310 (2)	142
C12*B*—H12*A*···F1*A*^iv^	0.95	2.52	3.289 (2)	138
C12*A*—H12*B*···F1*B*^v^	0.95	2.43	3.200 (2)	138
C15*B*—H15*A*···N1*B*^ii^	0.98	2.57	3.503 (2)	160

Symmetry codes: (i) −*x*+2, *y*, −*z*+3/2; (ii) −*x*+1, *y*, −*z*+3/2; (iii) *x*, −*y*+2, *z*−1/2; (iv) −*x*+2, *y*, −*z*+5/2; (v) −*x*+1, −*y*+2, −*z*+2.
